# Is the Dental Pulp Necessary in the Teeth of the Adult?

**Published:** 1901-05-15

**Authors:** James G. Palmer

**Affiliations:** New York


### IS THE DENTAL PULP NECESSARY IN THE TEETH OF THE ADULT?

BY JAMES G. PALMER, D.D.S., NEW YORK.

Extract of a paper read before the Central Dental Association of Northern New Jersey.

Reprinted from the *Items of Interest.*

In the abstract, possibly not. Some go even to the extent of saying that it is advantageous to remove the pulp, after a certain indefinite period in life, dispensing with it entirely. That which may be advantageous for some special cases does not seem to the writer to be indicated as a general practice.

The pulp has a definite function to perform. Is a period in human existence reached when it is not merely unnecessary, but actually useless, or, perhaps, even a menace? Evidently some think so, if their utterances have been understood.

What is the period of adult life when the pulp is no longer necessary? How is the time to be determined, if indeed it can be? The condition of the pulp in the teeth of one individual, at, say forty years of age, is entirely different from that of another individual of the same age.

Occasions may arise when devitalization of the pulp in sound teeth is advisable, and not always confined to adult life. Even in the teeth of quite young patients there may arise occasions for such devitalization. In the teeth of the adults extreme sensitiveness or other conditions of disease may be more successfully combated if the pulp be removed.

A dead tooth is not as strong as a live one, and is more liable to decay. The lines of cleavage are more marked, and if there is an extra leverage upon the tooth it is liable to fracture, to crumble away, as a live tooth will rarely do.

While the tooth is undoubtedly nourished by and through the pericementum, that is not sufficient for the entire tooth, else the friability referred to would not occur. The Almighty placed the pulp in a tooth for a specific purpose. Does that specific purpose end with some certain period more or less indefinite in human existence? I do not believe it.

The deciduous tooth is absorbed; the alveolus is absorbed; the pulp recedes; but does it, as a rule, become obliterated? When the occlusion is good; when the grinding surface has not worn away; does not the pulp, after a certain age, remain almost *in statu*

*quo?* Is it not in those cases where there has been much abrasion and undue strain that anything approaching obliteration is found? Those who view this matter from a purely theoretical standpoint may say otherwise, but my experience at the chair confirms this statement.

In the six anterior teeth, to the average dentist, there is, or should be, little difficulty in removing the pulp, thoroughly sterilizing and filling the canal; but in all the other teeth, bicuspids and molars, where fine tortuous canals are found, the possibility of trouble is greatly enhanced.

In connection with the treatment of pyorrhea alveolaris, some authorities favor the removal of the pulps in all teeth, of which the peridental tissues are seriously affected, with the idea of turning the pulp pabulum to the peridental membrane.

A conversation some time ago with one of these authorities, who has been understood to be quite in favor of this indiscriminate removal of the pulp, brought out the fact that he did not mean indiscriminate, but was so wrapped up in the beauties of it as he viewed it at the moment, that he did not see how far he was going, and so made more emphatic statements than he had intended. It is such statements that mislead men, particularly the younger practitioners, who are ever ready to take up a new idea when brought forward by a prominent man.

A good many years ago a man came out of the West with the startling statement that he would bore a hole in the jaw, set a tooth therein and have that tooth become firm and serviceable. We doubted. A prominent editor of one of our Eastern dental journals even stated that it was an utter impossibility because it was contrary to all physiological laws. Nevertheless it is accomplished every day.

In like manner the general removal of the pulp, possibly the indiscriminate removal, may be performed with less damage when all practitioners have been trained to far more delicate work than the majority of us are capable of at present, and will not make the mistake of destroying pulp after pulp when looking for some disturbance in the teeth which has an obscure origin.

I do not believe in the removal of the pulp upon slight provocation, and I do most emphatically believe that the dental pulp *is* necessary to the well-being of the teeth of the adult, except in rare cases.

### DISCUSSION.

DR. M. L. RHEIN: I limit the removal of unexposed pulps to cases which are likely to die after treatment. In the treatment of pyorrhea alveolaris it is of great value in many cases, while in others the loss of the pulp would not be helpful in the treatment and might be even detrimental. I would certainly oppose the obliteration of the pulp in every tooth subject to this disease. I would not say that the pulp which was nearly exposed by extensive caries should be destroyed; but we all fear the consequences of inserting permanent fillings in large carious cavities. I should not think of removing the pulp to cure sensitiveness of an exposed root. I should rather cut out the sensitive spot and insert a filling.

I would favor pulp extermination when there was from any cause absorption of the root, or a deposit of secondary dentin in the pulp substance, creating irritation. Again, I would favor it to divert the blood supply to an enfeebled peridental membrane, with the hope of building it up. The argument that pulps should never be removed when it is possible to retain them, because it is impracticable to thoroughly clean and perfectly fill the roots is not a good argument, for the reason that with present methods and skilled manipulators it is practicably possible to thoroughly clean or sterilize and fill the large majority of root canals.

Dr. J. Leon Williams: Thousands of treated pulpless teeth have accumulated sufficient evidence of the practicability of this operation and also furnishes the proof that the tooth pulp is not essential to the functional value of the tooth. Unlike the softer tissues, the teeth are passive organs of resistance, used in masticating food, and need only such active nutrition as is necessary to maintain a healthy relation to other contiguous vital structures. If it takes five years to effect a complete change in bone of average density it should require at least fifty years to completely renew the structure of dentin; so nutrition of this tissue dwindles to the vanishing point of importance.

A healthy pulp in a tooth insures the normal color and prevents possible pericemental irritation from infection. On the other hand, efforts to retain living pulp are often not only failures, but result in the death of the pulp and disastrous pericemental disease. In my judgment, it is better practice to sacrifice a healthy pulp which furnishes a doubtful prognosis, rather than to risk infection of the tooth tissue and peridental structures from a decomposing pulp, thus jeopardizing the future welfare of the tooth.

The whole question involves enlightened judgment. I am more and more convinced of the undesirability of attempting to save a pulp which has once been subjected to inflammatory action. Many pulps not actually exposed die after treatment and produce suppurative results, because the layer of dentin covering the pulps contained bacteria sufficient to cause these results after filling.

More systematic and scientific treatment should be adopted in the treatment not only of exposed pulps but infected dentin, especially in cases where there is any possibility of infection. When the pulp has been removed a more consistent and also persistent method of treatment should be employed than is usual, especially in questionable cases.

DR. I. N. BROOMELL: That the function of the pulp is largely generative can not be denied; this dentification continues with more or less regularity throughout the life of the organ, this function being more active in the used than idle teeth, resulting in continually thickening dentin and receding pulp. When this process has produced a sufficiently resistant tooth mass to withstand all possible force, the pulp looses its usefulness. Its loss, if necessary, is a matter of no great concern so long as its place is supplied with a properly adapted filling material.

The indiscriminate destruction of the dental pulp is not asked for; far from it, this can serve no good purpose. As long as the present imperfect methods of filling the pulp cavities are in vogue, the loss of the pulp in a certain percentage of cases will result more or less disastrously.

Replace the pulps of mature teeth in those cases which call for its removal with such substances and by such a method as will insure completeness somewhat approaching the natural conditions, and the longevity of the organ will not be affected.

DR. JNO. I. HART: The pulp is of immense advantage and should only be removed when it is the cause of direct or indirect reflex irritations, and not because it may in the future become the seat of morbid phenomena. The argument that pulps should be removed because they may give trouble would deprive some of us of our livers or kidneys. Our organs of speech should be removed also, because we have lived to regret some of our extravagant expressions of a few years since, when few were willing to admit the possibility of failure to save every pulp capped.

The author speaks of the destruction of pulps in "every case of pyorrhea," and the argument I have heard from a few gentlemen favoring this treatment is, that by cutting off the blood supply to the pulp, more is furnished to the pericementum. Temporarily, yes; permanently, no; and only in certain classes of cases do I find this treatment advantageous, but in the majority of instances I find the quality of the blood impaired rather than the quantity increased.

I do not revere the pulp simply because it is a part of the human economy, but because of the function it performs, and when your essayist asserted that its removal weakens the crown of a tooth, he stated a fact known to each one present. Conceding that many instances arise where its removal is absolutely necessary, (and here is where our ability as diagnosticians is tested to the utmost) I maintain that its removal, because the tooth is fully formed and requires it no more, is most fallacious. No less an authority than Dr. Louis Jack (in the "American Text-Book of Operative Dentistry," page 294), says:

"As the dental pulp by its supply of nutritive pabulum maintains the vitality of the dentin, and increases the resisting power of the tooth, it is important when this organ becomes exposed to agencies which threaten its destruction, to attempt its preservation when the conditions are favorable to that object. A further reason for maintaining the vitality of the dentin is that when the pulp becomes devitalized, the loss of cohesive force, which occurs as a consequence, leads sooner or later to the fracture and early loss of the tooth, the final result being delayed in proportion to the inherent strength of the tooth, and the period of life at which devitalization takes place," although in my estimation this risk may be modified by shortening the walls and protecting the same with some resisting filling material.

Dr. C. L. Hungerford: Is the dental pulp necessary in adult life? Certainly not; but it is unfortunate that it becomes necessary to add that this assertion does not mean that an attempt should be made to remove the pulp of every tooth that comes under your care. We only remove such pulps as stand in the way of expedient dental operations.

There is little doubt in my mind that thoughtful, observing dentists throughout the country have for years been in accord with the removal of dental pulps whenever they stood in the way of successfully supplying bridges, crowns or other treatment for the comfort or appearance of the teeth, only they lacked the courage to make public the assertion.

Every dentist who has had any experience will acknowledge that a pulpless tooth that has been well taken care of, will remain serviceable and comfortable for life. Why, then, we ask, should a patient who has lost a central incisor be asked to put up with some kind of a plate, or other frail or bungling contrivance when, by the destruction of the pulps in the sound adjacent teeth, a permanent bridge can be made that will defy detection, even by the patient himself?

After the removal of the enamel, dentin and pulp, there is no impairment of the functions of the remaining dental tissues, whereas, if these tissues were interdependent in the same way as the glandular system, the destruction of one would mean the impairment of all. The blood supply to the vascular or pulmonary system is a fair illustration. True bone is not seriously impaired by the removal of its medullary arteries and nerves.

Dr. R. R. Andrews: The wonderful network of anastomosing fibrils found in the dentin and pulp, which is their source of nervous and vascular supply, is certainly there for a permanent and vital purpose, and it does have an important function in conserving the health as well as the beauty of the teeth.

In my own investigation, I have found evidences that the dentin possesses the power of making the effort to protect itself from wear and infection not only in the teeth of the young, but on the grinding surfaces of the teeth of the aged, where the surfaces have been worn sometimes beyond the line of the original pulp cavity. This resistant action is one important point which should make the dental pulp seem necessary in the teeth of the adult. For as life advances, the grinding surfaces are sometimes worn away to the level of the gum; yet these teeth rarely decay. The surface which has undergone the change is darker in color than normal dentin, and by some may be taken for decay. But so far from being affected in this way, it is no longer liable to decay, except under very unfavorable circumstances. A tooth in this condition usually remains free from decay during life. It is my experience, that most of the teeth of healthy, robust persons retain their pulps in a healthy, active and vascular condition throughout life—and it was meant that they should. Secondary dentin is sometimes formed by the pulp as a protection, when it is irritated by the fracture of the tooth or by the thinning of the walls of the pulp cavity through excessive wear. There can be but little doubt that the pulp reacts and struggles against the invasion of infecting organisms, renders the progress of infection slower, and in favorable cases arrests decay altogether.

We must admit that there are thousands of pulpless, treated teeth that are doing excellent service. We must also admit that there are hundreds, equally as carefully treated, that have failed. We have all had this experience. Under certain pathological conditions, when the pulp is approached by invasion of organisms, when there are tissue lesions antagonistic to the health of the pericemental tissue, the extirpation of the pulp is the only proper practice to employ. In very many cases, I believe it is not employed as often as it should be. It is even proper to destroy the pulp of a healthy, live tooth, under certain conditions. But can any one rightfully assert that this pulpless tooth, filled with the best possible care, is in every way equally as perfect, as its wholly living and healthy mate? A wise provision of nature has supplied living fibrils from the pericemental membrane to and into the cementum, and when the work of extirpation of the pulp and its subsequent treatment and filling is rightfully performed, the operation, because of this living structure, may be eminently successful, and such a tooth may last for years. But in this tooth, the main mass of its organic structure is dead and but partly removed, and the tooth is thus liable to become opaque and discolored; the absence of a nourishing fluid, and the breaking up of the fibrilli probably account for this. There can be little attempt by the limited life the tooth still has, to resist the inroads of infection, or to give it normal color. Then, when the general vitality is lowered by constitutional disturbances, and the secretions become perverted, these pulpless teeth are usually the first to give pericemental trouble, and to break down.

Believing as I do that a live, healthy tooth is better than a pulpless one, no matter how carefully filled, and in the light of what evidence I have given you, strengthened by years of histological investigation, I must assert that I firmly believe that the dentinal pulp is necessary for the best possible conditions of the teeth in adult life; and I believe that we should strive to so conduct our practice that there should be the least possible need for its death and extirpation.

Dr. G. V. Black: I am of the opinion that the pulp of a tooth should never be destroyed except when conditions present which seriously threaten its vitality, either at once or in the near future. It is true the importance of the pulp decreases with age; but its influence over the health and effectiveness of the tooth never ceases. Many pulpless teeth render effective service for years, but they are not as effective, nor as safe, so far as the health of their membranes is concerned, as teeth having living pulps. I regard any tooth from which the pulp has been removed as seriously injured, although not passed giving reasonable service.

I have made some study of this with the knathodynamometer by trying the effective biting force of pulpless teeth and teeth with living pulps in the same mouth, and have uniformly found that, no matter how perfect their health seemed, their membranes were notably weaker than the membranes of neighboring teeth with living pulps.

Extreme care should be taken to save the life of pulps in the teeth of young people, especially before the age of twenty, for two prominent reasons: First, the smaller the apical foramen the more effective the root filling, and the better prospect for reasonable strength of the membranes, and continued health. Second, after the death of the pulp more or less diminution of the strength of the tooth, particularly of the dentin, is gradually occurring, which, in the event of a long life, seriously menaces the usefulness of the tooth.

Dr. E. A. Bogue, New York: In the discussion of scientific subjects, precision is necessary. As the question of the evening is far from precise, there can, of course, be no accurate discussion. "Is the living dental pulp necessary?" can be paralleled by, are two legs necessary?

It is evident that they are not necessary to life, nor to locomotion, nor dancing, as certain instances are known in which a man skates and dances, even though both his legs had been amputated. But it is not necessary to skate or dance; it is not absolutely necessary to walk.

If the intent of the question be, honestly, to ask whether the living dental pulp is of value, that question can easily be answered in the affirmative.

All writers of recognized ability, beginning with Dr. Black as the most recent, and going backward, acknowledge that the loss of the dental pulp brings in its train of sequences, brittleness, discoloration, and generally eventual abscess and loss, owing to the impossibility of sufficiently accurate manipulation in many of the minute roots of molar teeth to insure the absolute removal of all remnants of dead pulp, and the sealing of the pulp canals with some indestructible substance, clear to and not beyond the apex of the root.

The loss of the pulp, even in view of the aforementioned difficulties attending its treatment, and the impossibility of being sure in all cases that the operation will be successful, is much less serious than the loss of the tooth ; hence, it is better to lose the pulp than the tooth, and it is good surgery to undertake the removal of the pulp rather than the tooth. But it is bad surgery to unnecessarily remove any living tissue, that can be preserved.

